# Development of a prognostic gene signature based on an immunogenomic infiltration analysis of osteosarcoma

**DOI:** 10.1111/jcmm.15687

**Published:** 2020-08-21

**Authors:** Yiyang Yu, Hongliang Zhang, Tingting Ren, Yi Huang, Xin Liang, Wei Wang, Jianfang Niu, Yu Han, Wei Guo

**Affiliations:** ^1^ Musculoskeletal Tumor Center Peking University People’s Hospital Beijing China; ^2^ Beijing Key Laboratory of Musculoskeletal Tumor Beijing China; ^3^ Department of Orthopaedics the First Affiliated Hospital of Zhengzhou University Zhengzhou China

**Keywords:** bioinformatics, immunotherapy, osteosarcoma, prognostic

## Abstract

Osteosarcoma is the most common primary malignant bone tumour predominantly occurring in children and adolescents with a high tendency of local invasion and early metastases. Currently, tumour immune microenvironment (TME) is becoming the focus of studying of malignant tumours.. However, no sound evidence shows a specific immune molecular target in osteosarcoma. We downloaded the gene expression profile and clinical data of osteosarcoma from the TARGET portal, and extracted and normalized via R software. Then, the immune cell infiltration assessed by CIBERSORT and ESTIMATE algorithms. Three survival‐related immune cells and immune score were obtained via Kaplan‐Meier survival analysis, and 232 immune‐related genes were obtained as candidate genes. Enrichment and protein‐protein interaction co‐expression analyses were performed to identify 13 hub genes. Lastly, a seven gene prognostic signature was identified by univariate and multivariate Cox regression analyses. More importantly, our validations and TIMER algorithm suggested this immune‐related prognostic signature a good predictive tool. Our findings have provided novel insights that could demonstrate new targets of immunotherapy in osteosarcoma.

## 1 INTRODUCTION

Osteosarcoma is the most common primary malignant bone tumour predominantly occurring in children and adolescents with a high tendency of local invasion and early metastases. Currently, the systemic chemotherapy plus wide surgical resection are generally recognized as the most effective therapy for this malignancy. However, the main protocol has not been improved since 1980s. And neither does the cure rate.

The investigations of prognostic factors and biomarkers of cancer and sarcoma are in progress for these years, with the help of the next‐generation sequencing (NGS), single‐cell sequencing (SCS) and analysis techniques. Several studies on osteosarcoma reported that tumour size, metastatic disease at the time of diagnosis, histological grade, histologic response to neoadjuvant chemotherapy and adequate surgical margins have consistently shown a strong correlation with prognosis and outcome.^1^, ^2^ Additionally, many biomarkers have been identified to be correlated with the progression and tumorigenesis of osteosarcoma, such as MYC and microRNA‐133a.^3^, ^4^, ^5^ But the rarity and heterogeneity of the tumour itself represent a limitation in the development of practical prognostic factors and biomarkers. Currently, the emerging researching focus of malignancy is tumour microenvironment (TME), which represents the communication between tumour cells and the corresponding immune cells. It mirrors the traits of a tumour from another perspective. And several molecular mechanisms are potentially being developed into immune therapeutic targets. Though there were many clinical trials of immune therapies conducting on osteosarcoma, no sound evidence shows a specific molecular target effective.^6^, ^7^, ^8^ It still remains a big unknown world to be explored.

The tumour‐infiltrating immune cells play an important role in TME.^9^ Many algorithms were exploited to assess the infiltration of immune cells in genetic levels, such as Cell type Identification By Estimating Relative Subsets Of RNA Transcripts (CIBERSORT), ESTIMATE, Tumor Immune Estimation Resource (TIMER), ImmPort and xCell. In this study, we provide a new approach combining some of these algorithms together to assess immune cell infiltration of osteosarcoma samples for the first time. The RNA‐seq gene expression profile and clinical data were acquired from the Therapeutically Applicable Research To Generate Effective Treatments (TARGET) database, which is the most comprehensive genomic resource for childhood cancers and sarcomas. Moreover, our study then provides the cell and gene signature that could be considered biomarkers for prognosis for osteosarcoma immune therapy. And it might shed some light on identifying a new potential immune target for osteosarcoma.

## 2 MATERIALS AND METHODS

### 2.1 Study design and data collection

**FIGURE 1:**
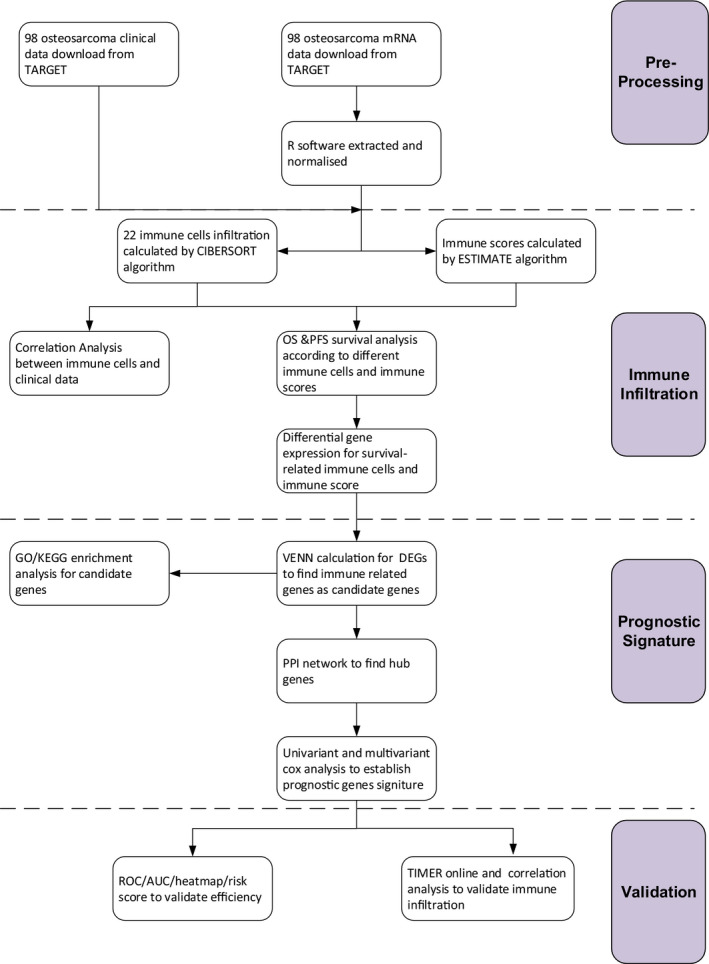
Flow chart of the study design. TARGET, Therapeutically Applicable Research To Generate Effective Treatments; CIBERSORT, Cell type Identification By Estimating Relative Subsets Of RNA Transcripts; ESTIMATE, Estimation of Stromal and Immune cells in Malignant Tumor tissues using Expression data; OS, overall survival; PFS, progression‐free survival; GO, Gene Ontology; KEGG, Kyoto Encyclopaedia of Genes and Genomes; DEG, differentially expressed gene; PPI, protein‐protein interactions; ROC, receiver operating characteristic; AUC, area under curve; TIMER, Tumor Immune Estimation Resource

The raw expression data and clinical data were downloaded directly from TARGET (OS) database including 98 osteosarcoma patients with 101 samples. The RNA‐seq data were updated on 4 September 2018. And the clinical data were updated on 5 August 2019. Then, R software (“tximport” and “edgeR” packages) was used to extract raw data and sort to obtain gene expression matrixes. The study design was shown in a flow chart (Figure 1), and the clinical data were arranged in Table S1. The validation cohorts were downloaded from GEO^10^ in the National Center for Biotechnology Information database. The gene expression profiles of GSE21257, GSE39058 and GSE16091 met our validation conditions.^11^, ^12^, ^13^

### 2.2 CIBERSORT, ESTIMATE and TIMER

Cell type Identification By Estimating Relative Subsets Of RNA Transcripts (CIBERSORT) is an analytical tool developed by Newman to assess immune cell infiltration ratio based on RNA‐seq profiles.^14^ We ran CIBERSORT locally in R software. The abundance ratio matrix of 22 immune cells was obtained with *P* < 0.05. Estimation of Stromal and Immune cells in Malignant Tumor tissues using Expression data (ESTIMATE) algorithm is developed by Yoshihara K to calculate the immune score of sequencing samples.^15^ Furthermore, the osteosarcoma samples were divided into high‐ or low‐score groups to identify a possible association of immune score with overall survival and progression‐free survival. TIMER (https://cistrome.shinyapps.io/timer/) is a comprehensive resource to systematically analyse immune infiltrates.^16^ The prognostic signature was validated for immune correlation via TIMER.

### 2.3 Clinical relationship with survival‐related immune cells

The data of metastasis, relapse, necrosis stage and 5‐year survival were analysed in the study using Wilcoxon rank‐sum test. The definitions of clinical traits were based on the osteosarcoma clinical trials.^17^ “Relapse”: confirmed primary relapse cases in 5 years; to delete the unconfirmed alive cases followed less than 5 years. “Necrosis stage”: histological necrosis rate < 90 as Stage 1/2; histological necrosis rate ≥ 90 as Stage 3/4. “5‐year survival”: cases with follow‐up time more than 5 years. All the cases were summarized in Table S1. Differences among clinical parameters were tested using independent *t* tests. *P*‐values of less than 0.05 were considered statistically significant.

### 2.4 Differentially expression gene analysis and VENN calculation

Differentially expression genes (DEGs) were screened via the R software edgeR package (http://bioconductor.org/packages/edgeR/) setting a false discovery rate (FDR) < 0.05 and a log2 |fold change|> 1 as the cut‐off values. 4 sets of DEGs were obtained according to the survival‐related immune cell infiltration level or the ESTIMATE immune score level. All sets of DEGs were then calculated via a VENN map online tool (http://bioinformatics.psb.ugent.be/webtools/Venn/). The most overlapping region was selected as candidate genes.

### 2.5 Gene ontology and Kyoto encyclopaedia of genes and genomes enrichment analyses

Gene Ontology (GO) and Kyoto encyclopaedia of genes and genomes (KEGG) pathway analysis were conducted via R software “clusterProfiler” package (http://www.bioconductor.org/packages/release/bioc/html/clusterProfiler.html). Counts ≥ 4 and *P* < .05 were set as the enrichment cut‐offs to screen meaningful enrichment results. P‐value was the judgement of significance of enrichment results. The enrichment results were visualized via the ggplot2 R package.

### 2.6 Protein‐protein interaction network construction and MCODE to identify hub genes

The STRING database (https://string-db.org/) was used to explore protein‐protein interaction (PPI) network construction of 232 candidate genes, and the combined score was set to ≥ 0.4. Then, the network was reconstructed via Cytoscape software “molecular complex detection (MCODE)” plug‐in. The degree cut‐off was set to 2, and node score cut‐off: 0.2. The most significant module was screened (score > 10, Num.nodes > 50), and the genes were identified as Hub genes.

### 2.7 Survival analysis

The Kaplan‐Meier analysis for overall survival (OS) and progression‐free survival (PFS) was proceeded. PFS was calculated based on the “First Event” in clinical data Table S1. The survival analysis was applied with the aid of R software, and the log‐rank was utilized to test. We identified survival‐related immune cells according to the results of the Kaplan‐Meier survival analysis. The analysis also applied on 13 hub genes.

### 2.8 Human clinical specimens

17 primary osteosarcoma samples (In situ) with 5 paired adjacent normal tissues and 7 paired lung metastases samples were used in this study for immunohistochemical analysis. All tumour specimens mentioned in this study were acquired from the Musculoskeletal Tumor Center, Peking University People's Hospital (Beijing, China). Informed consent was obtained from each patient and their guardians if patients were under 18 years old, and the study was approved by the ethics committee of Peking University People's Hospital.

### 2.9 Immunohistochemistry

After deparaffinating, rehydration and heat‐induced antigen retrieval with citrate solution, paraffin sections were incubated with the corresponding antibodies overnight at 4°C. The staining scores were the sum of the staining area (0: no staining or staining in <10%, 1: staining in 10%–40%, 2: staining in 40%–70%, 3: staining in ≥70%) and the staining intensity (0: no staining, 1: yellow, 2: brown, 3: maroon). The immunohistochemistry (DAB) was assessed by two independent pathologists without any previous information of the clinical characteristics and outcomes. The antibodies are listed in Table S4.

### 2.10 Statistical analysis

13 hub genes were submitted for univariate and multivariate CoxPH regression analyses. Seven immune‐related genes were finally included in the risk prognostic model. The risk score is calculated as ∑ coefficients * expression values. AUC of the survival ROC curve was calculated via the survival ROC R software package to validate the performance of the prognostic signature.^18^

R software was used for most of the bioinformatics and statistical analyses in this study including RNA‐seq data normalization and transformation, CIBERSORT and ESTIMATE, differential gene expression analysis, CoxPH and KM survival analyses, ROC analysis, as well as Spearman rank correlation analysis.

Student's *t* test was used to analyse the data for two groups. All data were analysed using GraphPad Prism 8.0.2 (GraphPad software, Inc, La Jolla, CA, USA). *P*‐value < 0.05 was considered statistically significant.

## 3 RESULT

### 3.1 Identification of survival‐related immune cells

**FIGURE 2:**
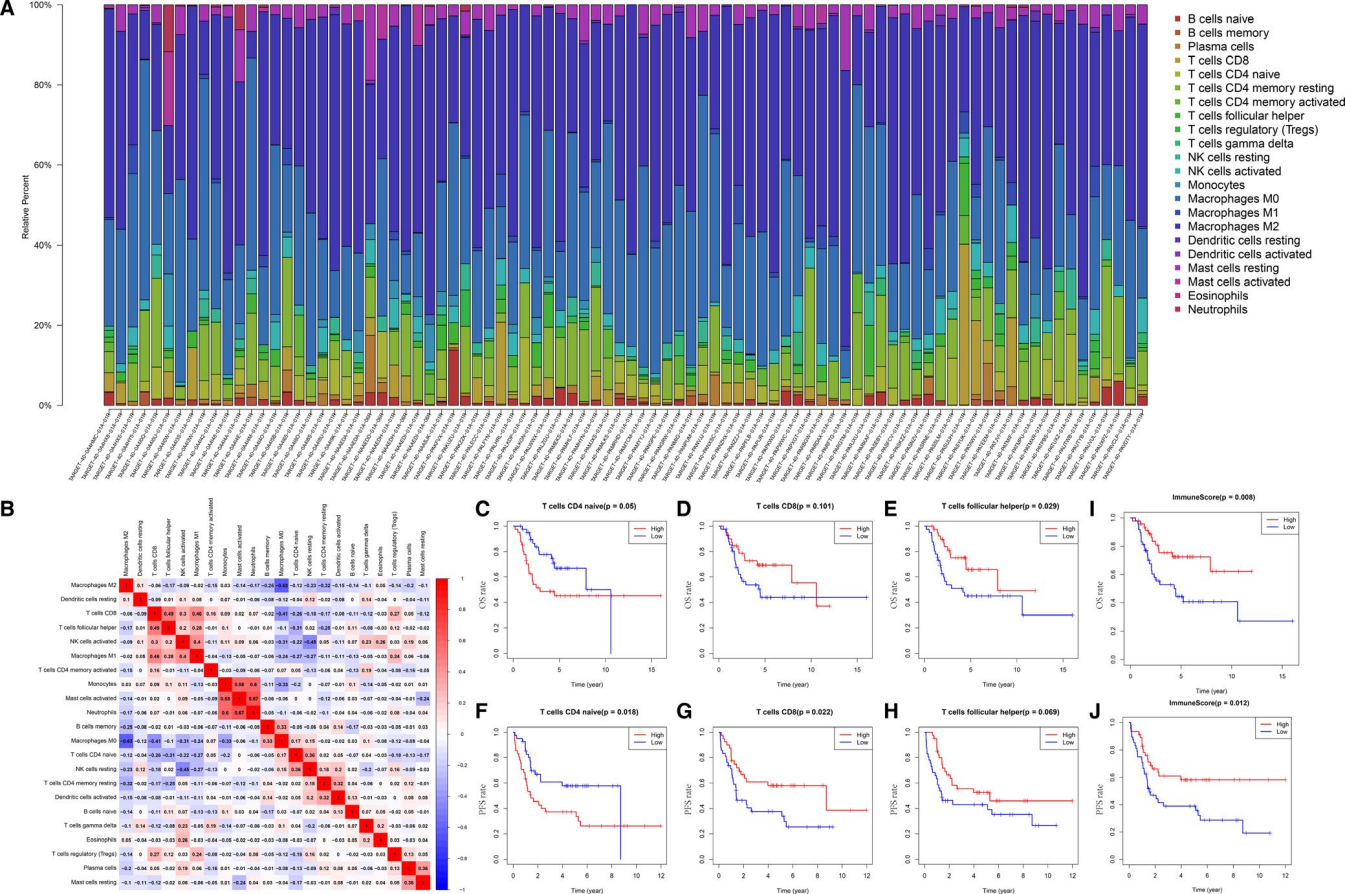
CIBERSORT and ESTIMATE algorithms applied to identify survival‐related immune cells and to find relationship between immune score and OS or PFS survival. (A) The 22 immune cell infiltration of osteosarcoma samples assessed by CIBERSORT algorithm. (B) The correlation analysis of all the immune cells. (C‐H) The OS and PFS survival analysis of the three immune cells. (I‐J) The survival analysis of ESTIMATE immune score

The infiltration ratio of 22 immune cells calculated by CIBERSOT algorithm and their correlations is shown in Figure 2A, B. In osteosarcoma samples, M0 macrophage and M2 macrophage are the major constituent of infiltration immune cells. Interestingly, M0 macrophage shows negative correlated with M2 macrophage in the correlation analysis. We also find that CD8^+^ T cell, follicular helper T cell and M1 macrophage are significantly positively correlated. And monocyte, activated mast cell and neutrophils are positively correlated. Then, we analysed the relationship between infiltration immune cells and overall survival or progression‐free survival via Kaplan‐Meier analysis (shown in Figure 2C‐H). We finally identified T‐cell follicular helper, T‐cell CD4 naive and T‐cell CD8 related to survival significantly.

### 3.2 Relationship between ESTIMATE immune score and OS or PFS

All of 98 samples were assessed by ESTIMATE algorithm in R software. According to the immune score, we separated them into 2 groups (50 in high group, 48 in low group) in order to do survival analysis next. Both OS and PFS Kaplan‐Meier curves reveal that the high immune score group is significantly associated with better survival rate (OS *P*‐value = 0.008; PFS *P*‐value = 0.012 Shown in Figure 2I, J).

### 3.3 Survival‐related immune cells were correlated with clinical data

**FIGURE 3:**
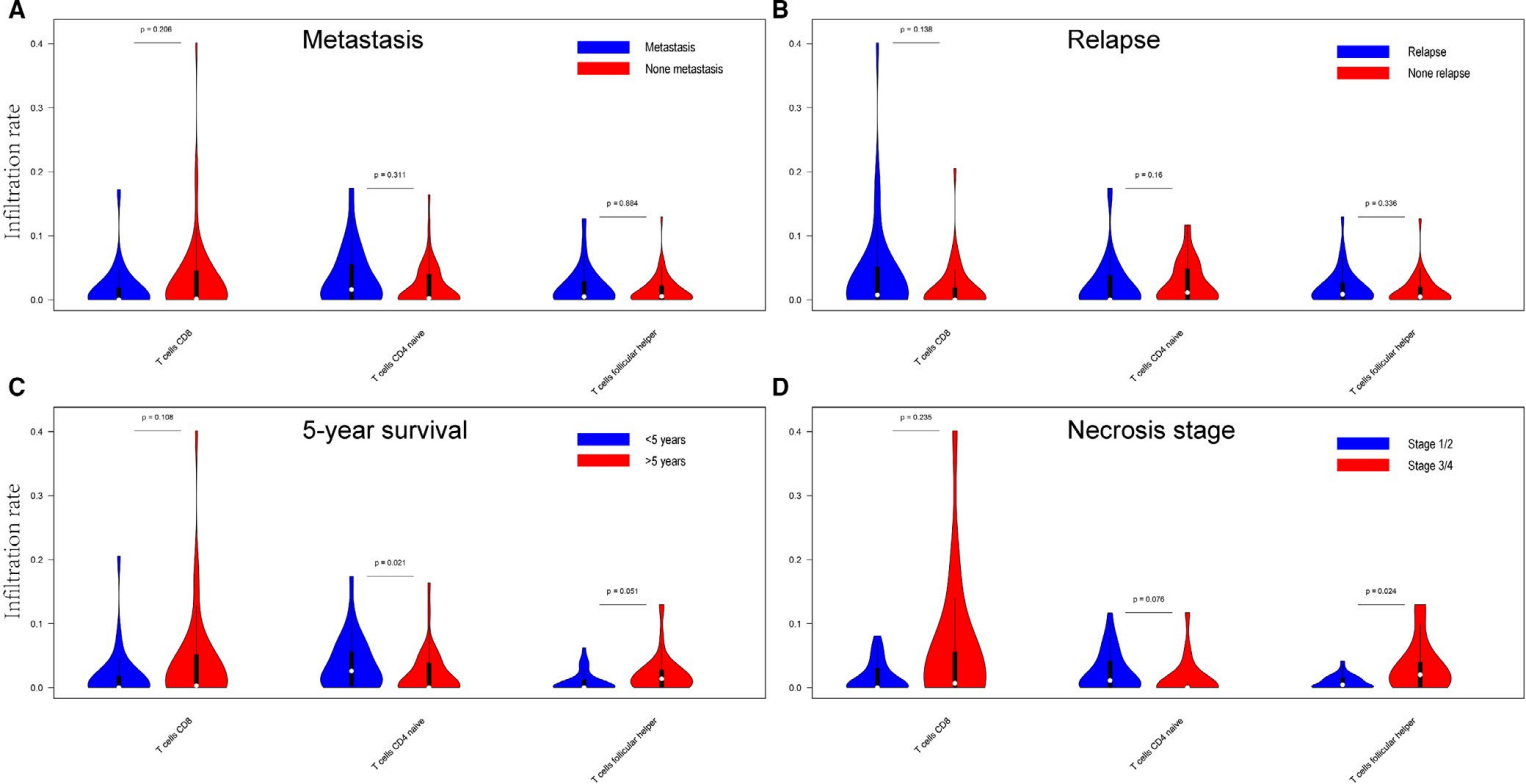
The relationship between survival‐related immune cells and clinical data. The long‐time survival (>5 y) rate was correlated with high infiltration of T‐cell follicular helper and T‐cell CD8. AND better necrosis condition was associated with high infiltration of T‐cell follicular helper. (A‐D) The relationship between immune cell infiltration and metastasis, relapse, 5‐year survival and necrosis stage

Our correlation analysis between 3 survival‐related immune cells and clinical traits (metastasis, relapse, necrosis stage and 5‐year survival) shows that long‐time survival (>5 years) rate was correlated with high infiltration of T‐cell follicular helper and T‐cell CD8 (*P*‐value = 0.051 and 0.108), and low infiltration of T‐cell CD4 naive (*P*‐value = 0.021). It also illustrate that better necrosis condition was associated with high infiltration of T‐cell follicular helper and T‐cell CD8 (*P*‐value = 0.024 and 0.235), and low infiltration of T‐cell CD4 naive (*P*‐value = 0.076). There were no significant differences in Metastasis and Relapse conditions. The result is shown in Figure 3.

### 3.4 Identification of differentially expressed genes of survival‐related immune cells and ESTIMATE immune score

**FIGURE 4:**
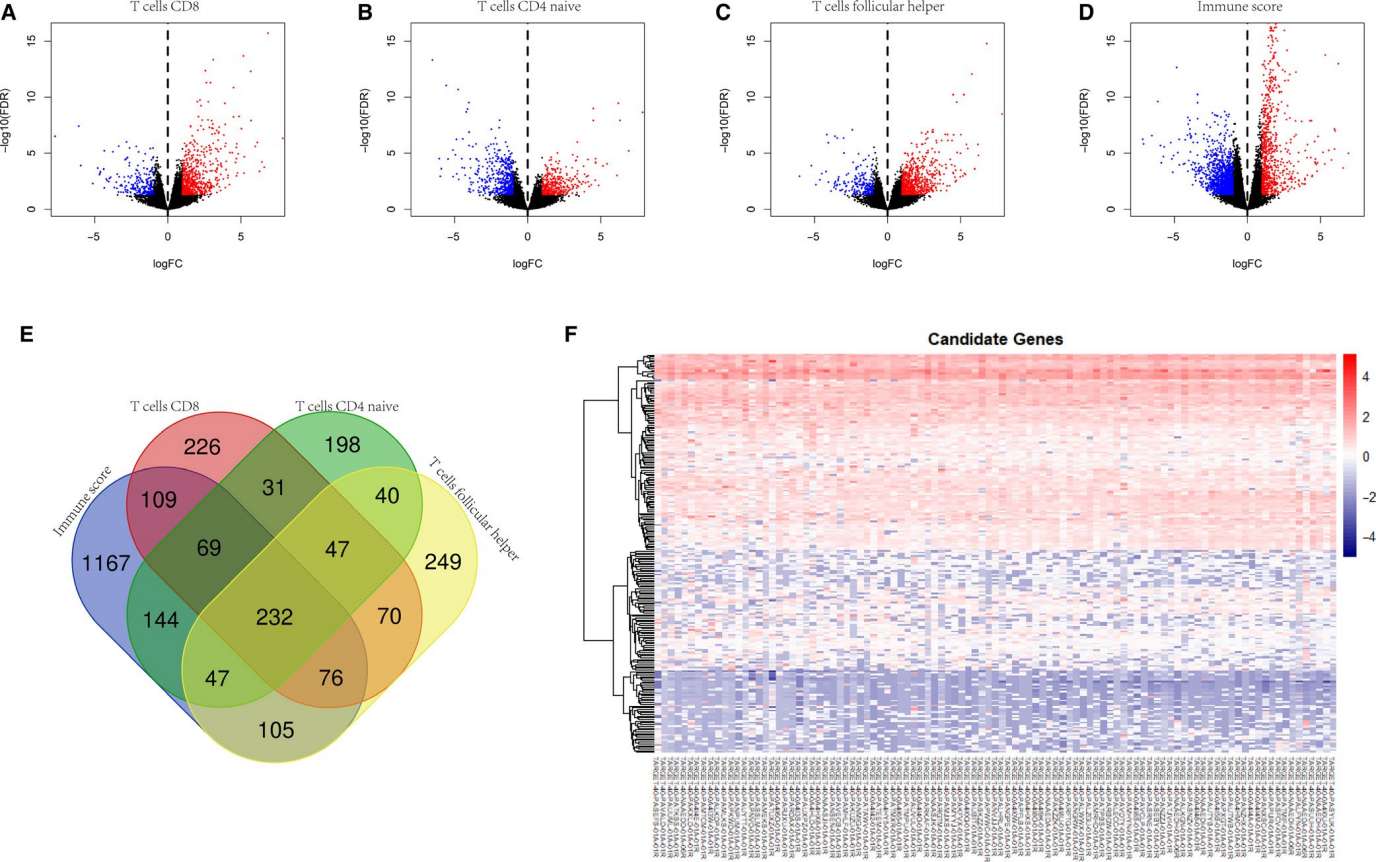
Identification of candidate genes. (A‐D) Volcano plots demonstrating DEGs between high and low infiltration of CD8 + T cells, CD4 + naïve T cells, follicular helper T cells and immune score. Red dots represent up‐regulated genes, and blue dots represent down‐regulated genes. (E) VENN calculation applied to identify 232 candidate genes. (F) Heatmap of 232 candidate genes in all osteosarcoma samples

4 sets of DEGs were extracted in R software according to the survival‐related immune cells infiltration and the ESTIMATE immune score. There were 860 genes related to T‐cell CD8, 808 to T‐cell CD4 naive, 866 to T‐cell follicular helper and 1949 to ESTIMATE immune score. The volcano plots of DEGs were shown in Figure 4A‐D. Then, we calculated all the DEGs in VENN map analysis (Figure 4E). 232 genes were selected as candidate genes. As shown in the heatmap (Figure 4F), the expression profiles of these genes were relatively consistent among these 101 samples.

### 3.5 GO enrichment analysis and KEGG pathway analysis of DEGs

**FIGURE 5:**
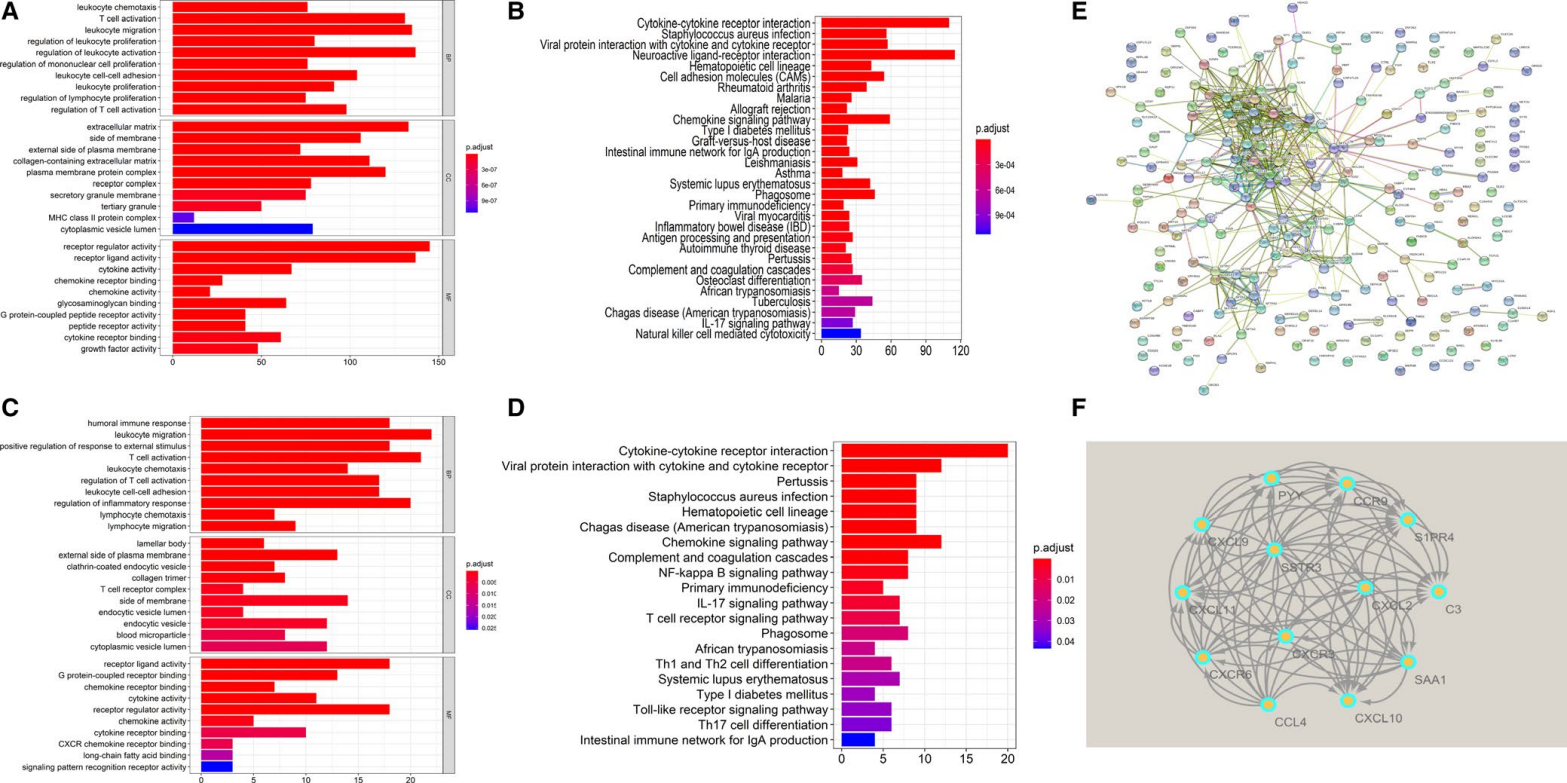
Gene functional enrichment and PPI network to identify hub genes. (A‐B) The results of GO enrichment and TOP20 KEGG pathway of all 2810 DEGs. (C‐D) The results of GO enrichment and TOP20 KEGG pathway of 232 candidate genes. (E) Protein‐protein interaction network of 232 candidate genes. (F) The most significant module of 13 hub genes in the Cytoscape MCODE plug‐in

GO and KEGG analyses of all 2810 DEGs and 232 candidate genes were performed via R software “clusterProfiler” package. Go analysis of 2810 DEGs showed that “leukocyte chemotaxis”, “T cell activation”, “leukocyte migration” were most frequently enriched among biological processes, “extracellular matrix”, “side of membrane”, “external side of plasma membrane” among cellular components, and “receptor regulator activity”, “receptor ligand activity”, “cytokine activity” among molecular functions. For the KEGG analysis, “Cytokine‐cytokine receptor interaction”, “Staphylococcus aureus infection” and “Viral protein interaction with cytokine and cytokine receptor” were most often enriched. As expected, the results of GO and KEGG analysis of 232 candidate genes were very similar to the results of 2810 DEGs. 45% results were same (Figure 5A‐D).

### 3.6 Identification of hub genes

The PPI network of 232 candidate genes mapped by STRING was shown in Figure 5E. There were 162 nodes and 590 edges in this network. Then, the Cytoscape MCODE plug‐in was used to reconstruct the PPI network. 7 modules were analysed, and the most significant module was screened (Figure 5F). 13 genes in the module were identified as Hub genes for the next analyses, which were CXCR3, SSTR3, SAA1, CCL4, PYY, CCR9, CXCL9, CXCL11, C3, CXCL2, S1PR4, CXCL10 and CXCR6 (Table S2).

### 3.7 Exploration of immune‐related prognostic gene signature model and validation

**FIGURE 6:**
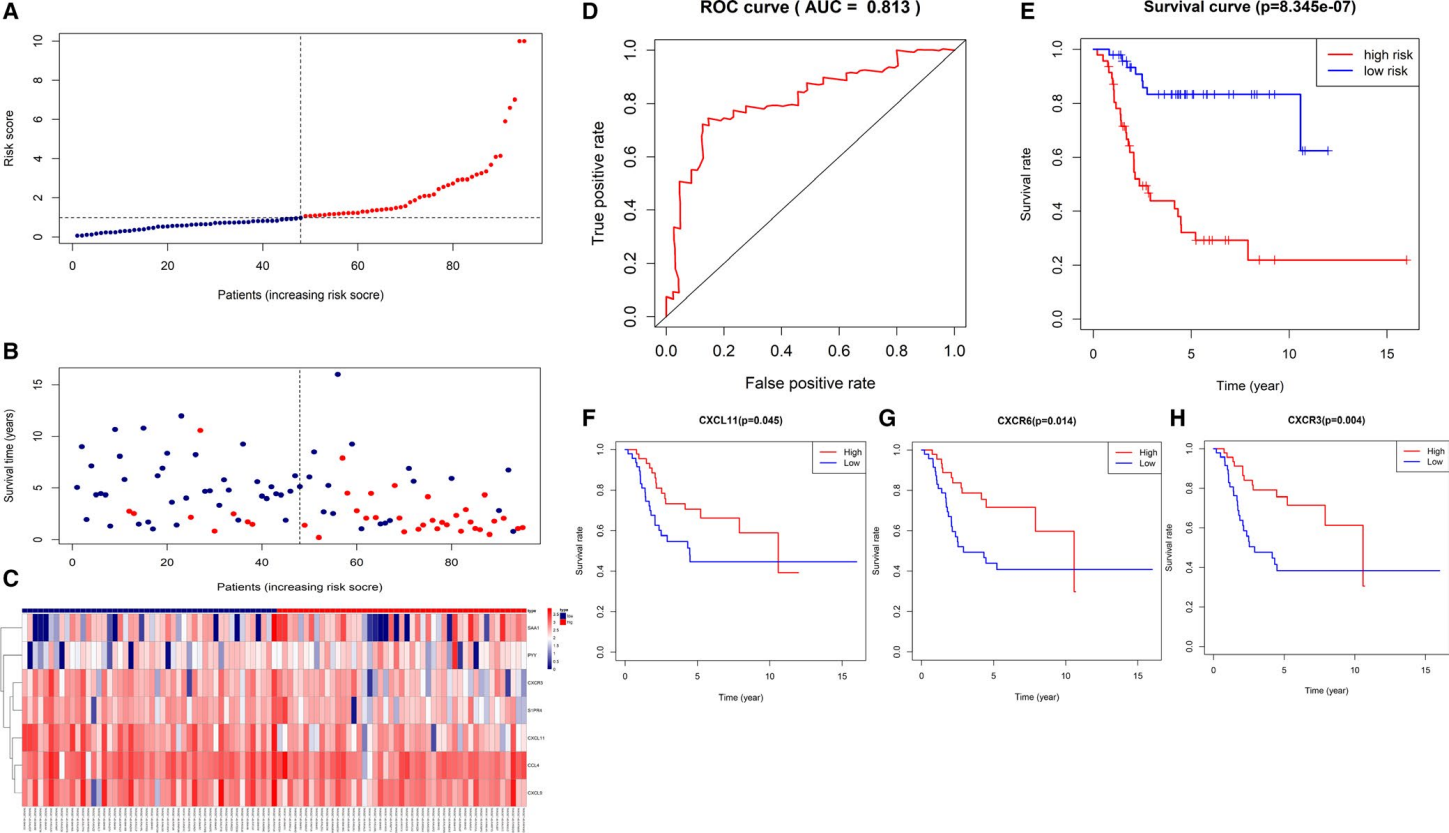
Exploration of immune‐related prognostic gene signature model and validation. (A‐C) The distribution of the survival status, risk scores and expression of 7 genes in the prognostic signature. (D) Receiver operating characteristic (ROC) curve and the area under curve (AUC). AUC = 0.813 shows moderate potential for this prognostic signature. (E) Survival analysis of risk score. High‐risk score was significantly associated with poor outcome (*P* < 0.01). (F‐H) Survival analysis of 13 hub genes. CXCL11, CXCR6 and CXCR3 are independently associated with overall survival. CXCL11 and CXCR3 are included in the prognostic signature

To identify key components of the hub genes, univariate and multivariate Cox regression analyses were further applied. All the 13 genes were included in both univariate and multivariate Cox regression analyses. The univariate result was shown in Table S3. In multivariate Cox regression, seven genes were finally included in the prognostic signature with a minimum Akaike information criterion (AIC) value of 290.64 estimated by R software “survival” package. The formula of risk score was as follows: Risk score = Expression level of CXCR3 * (−0.2888) + Expression level of SAA1* (0.1504) + Expression level of CCL4* (0.2079) + Expression level of PYY* (0.2266) + Expression level of CXCL9* (0.4665) + Expression level of CXCL11* (−0.4468) + Expression level of S1PR4* (−0.3696). The distribution of the survival status, risk scores and expression of the 7 genes is illustrated in Figure 6A‐C. Then, receiver operating characteristic (ROC) curve was performed to validate the diagnostic role of the signature. The area under curve (AUC) of the ROC curve was 0.813, suggesting moderate potential for this prognostic signature in survival monitoring (Figure 6D). And the survival analysis revealed that the high‐risk score group was significantly associated with poor outcome (OS, *P* < 0.01), which suggested this immune‐related prognostic signature a good predictive tool (Figure 6E). Moreover, Kaplan‐Meier survival curves of OS were then applied on all 13 hub genes. The results showed that high expression of CXCL11 (*P* = .045), CXCR6 (*P* = .014) and CXCR3 (*P* = .004) predicted poor OS (Figure 6F‐H). The similar results were shown in our validation cohorts (Figures S2A‐E, S3A‐E, S4A‐E).

### 3.8 Validation of the correlation of the prognostic signature model and immune cells infiltration

**FIGURE 7:**
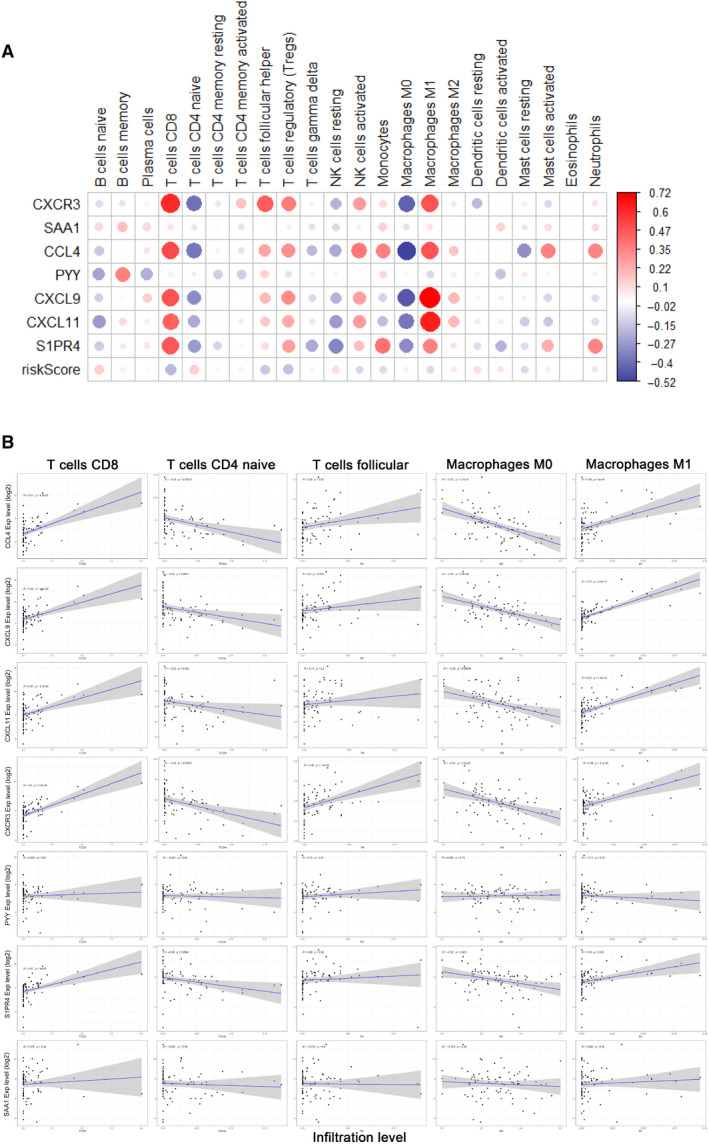
The validation of correlation analysis of prognostic signature and immune cells infiltration. (A) The correlation analysis. The x‐axis represents immune cell types, and y‐axis represents genes. Red dots represent positive correlation, and blue dots represent negative correlation. The dot size represents P‐value. The bigger the dot is, the smaller the P‐value is. And shade of colour represents Pearson correlation index r. (B) The definite correlationships between genes in the prognostic signature and infiltration of CD8 + T cells, CD4 + naïve T cells, follicular helper T cells, M0 and M1 macrophages. The correlation was performed by using Pearson correlation analysis. Each dot represents a sample, and the blue line represents the relationship between the expression level of each gene and immune cell infiltration level

Next, the method of Pearson correlation analysis was applied to validate the relationship between the prognostic signature and the infiltrating immune cells. The results between the 7 genes and 22 infiltrating immune cells were shown in Figure 7A. As shown in Figure 7B, the prognostic genes were significantly correlated with the infiltration of CD8 + T cells, CD4 + naïve T cells, follicular helper T cells, M0 and M1 Macrophages. The expression profiles of gene CXCR3, CXCL11 and CCL4 mainly reflected the infiltration of the survival‐related immune cells which calculated by CIBERSORT. Besides, TIMER was also applied to validate the infiltration of the immune cells on TCGA‐SARC data set (Figure S1). And the same methods were used in validation cohorts (Figures S2F‐G, S3F‐G, S4F‐G).

### 3.9 CXCL9, CXCL11/CXCR3 axis validation

The IHC assays were performed on our own samples to validate the expression level of CXCL9, CXCL11/CXCR3 axis (Figure S5). We found that IHC staining exhibits that all the protein expression of CXCL9, CXCL11/CXCR3 axis was significantly elevated in osteosarcoma tissues compared with adjacent normal tissues (bone, marrow, soft tissue and cartilage). Meanwhile, we compared the protein expression of CXCL9, CXCL11/CXCR3 axis in paired lung metastasis samples (Figure S6). This results showed that no significant differences between metastasis and in situ lesions.

## 4 DISCUSSION

Osteosarcoma is the most common primary malignant bone tumour with poor clinical outcome. Though the immunotherapy for cancers and sarcomas is rapidly developed, a comprehensive study on osteosarcoma immunogenome characteristics has been rarely conducted. Plenty of clinical studies illustrate that cancer immunotherapy is potential to play a key role in future clinical cancer management.^19^ This study aims to identify and explore cells and genes closely related to immune infiltration and clinical outcomes. We also provide a comprehensive, integrated analysis approach to acquire an individualized immune prognostic signature proposing to reflect immune cell infiltration and clinical survival outcomes of osteosarcoma patients.

CIBERSORT, ESTIMATE and TIMER are widely approved algorithms to assess immune infiltration and scores based on RNA‐seq profiles. In this study, these algorithms are synthetically applied to process osteosarcoma sequencing data acquired from TARGET data set. Our recombined approach could help with more accurate and highly correlated gene signature predicting clinical outcome.

In this study, the immune cell infiltration landscape of osteosarcoma samples was obtained via CIBERSORT algorithm. Compared with other immune cells, macrophage (M0, M1 and M2) is the majority infiltration in osteosarcoma, especially M0 and M1. But we did not find it correlated with OS or PFS survival. It has been reported that tumour‐associated macrophage (TAM) plays an important part in osteosarcoma immune environment.^20^ A study by Buddingh et al demonstrated that the expression of TAM‐related genes was connected with metastasis.^11^ Dhupkar P et al illustrated that the change in macrophage phenotype from M2 to M1 induces regression of lung metastasis.^21^ Though it remains controversial to determine that TAM is supportive or suppressive on solid tumours, it is worthy further investigations to focus on the dynamic change in TAM infiltration in osteosarcoma. The next survival analysis of immune cell infiltration showed that T cell CD8, T cell CD4 naive and T‐cell follicular helper were related to the OS or PFS of patients with osteosarcoma. In spite of relatively little proportion of CD8 + T cell infiltrated, it obviously impacts the survival outcome. As it has confirmed in other solid tumours, higher infiltration of CD8 + T cell always predicts higher survival rate.^22^, ^23^ In recent years, some investigations proposed that immune checkpoint blockage such as CTLA‐4 and PD‐L1 inhibits progression of osteosarcoma by stimulating CD8 + T cell.^24^, ^25^, ^26^ Cytotoxic lymphocyte (CTL) activation of CD8 + T cells could lead to less lung metastasis and suppression of osteosarcoma progression. Another survival‐related immune cell is naïve CD4 + T cell, also known as CD45RA + T cell. It is correlated with poor outcome in our study. Naive T cells were previously thought to traffic exclusively to lymphoid organs. But it is recently documented that they can be detected in non‐lymphoid organs, even in inflamed tissues and in tumours.^27^, ^28^, ^29^, ^30^ Shicheng Su et al reported the abundance of naive CD4 + T cells and T‐regs is closely correlated, both indicating poor prognosis for breast cancer patients. It may be attractive strategy for anticancer immunotherapy to inhibit naive CD4 + T‐cell recruitment into tumours by interfering with PITPNM3 recognition of CCL18.^31^ Follicular helper T cells were also associated with poor outcome in our study. Gao Wenwu et al revealed the IL‐21 production by follicular helper T cells was significantly reduced in the presence of PD‐L1 osteosarcoma cells and was rescued by the anti–PD‐L1 antibody.^32^ However, there is little evidence that follicular helper T cells are correlated with survival time in osteosarcoma. In addition, immune score of ESTIMATE algorithm was applied to assess the osteosarcoma samples in our analysis. It was shown that high immune score was significantly associated with poor outcome. Combination of the results from these two algorithms could describe the immune status more comprehensively.

To further confirm the correlationship between these 3 survival‐related immune cells and clinical traits (metastasis, relapse, necrosis stage and 5‐year survival), a correlation analysis was then conducted. Our result suggests that the immune cell infiltration in osteosarcoma could directly impact the 5‐year survival rate, and it might impact the overall survival rate via influencing the tumour necrosis stage. While there are no significant differences in metastasis and relapse conditions in our study, it is not against the theory that immune cells are involved in metastasis and relapse in osteosarcoma. This means that in our study the survival‐related immune cells impact the prognosis but not primarily by influencing metastasis and relapse. Metastasis and relapse are so complicated pathological processes in osteosarcoma that many experiments have illustrated the immune microenvironment takes part in.^33^, ^34^ We support that it will be more credible if the study samples were paired with metastasis or relapse lesions. In line with previous reviews,^17^, ^35^, ^36^ poor necrosis stage (<90% necrosis) was potentially responsible for the poor overall survival of patients with osteosarcoma. As we know, poor necrosis stage always represents a poor histological response to chemotherapy. The Huvos grade (1‐2, <90% necrosis; 3‐4, >90% necrosis) was always included in prognostic factors for osteosarcoma.^37^

The GO and KEGG enrichment analysis were conducted to describe the relationship among the DEGs. As we expect, the results of all the DEGs and candidate genes calculated by VENN were highly similar. It is supposed that the 232 candidate genes were dominant in the gene functional enrichment analysis. Of the KEGG results, cytokine‐cytokine receptor interactions and chemokine signalling pathways were mainly involved. Interestingly, the similar result was reported in an immunogenomic analysis of papillary thyroid cancer.^38^ Our results also suggest that NF‐kappa B signalling pathway and IL‐17/TH17 signalling pathway involved in regulating survival‐related immune cell infiltration in osteosarcoma. The general consensus is that NF‐kappa B signalling activation maintains tumour cell survival to promote osteosarcoma progression, invasion and metastasis.^39^, ^40^ Trieb Klemens et al illustrated that the expression of receptor activator of nuclear factor kappa B (RANK) was immunohistochemically evaluated in biopsies of high‐grade osteosarcoma, and was found to be correlated with histological response to chemotherapy and overall survival.^41^ NF‐kappa B provides a mechanistic link between inflammation and cancer, and is a major factor controlling the ability of both pre‐neoplastic and malignant cells to resist apoptosis‐based tumour surveillance mechanisms.^42^ Navet Benjamin et al reported inhibition of nuclear factor kappa B signalling was capable of reversing the metastatic effect favoured by RANK expression in osteosarcoma cells in an immune‐compromised context.^43^ IL‐17/TH17 signalling pathway is another notable result. Th17 cells are a specialized subset of CD4 + T cells that are essential in driving inflammation and infection through a signature cytokine IL‐17. Wang Mingmin et al found that serum IL‐17A was higher in osteosarcoma patients with metastasis and IL‐17A/IL‐17RA interaction promoted the metastasis of OS in nude mice.^44^ A recent systemic review based on published results showed that IL‐17 is associated with poor prognosis in cancer while Th17 cells are not.^45^ It is widely acknowledged that Th17 cells and IL‐17 promote inflammation via production of pro‐inflammatory molecules. However, their role in cancer and sarcoma is still inconclusive. Other signalling pathways, such as T‐cell receptor, Toll‐like receptor, complement and coagulation cascades, are more or less enriched in our results.

13 hub genes were identified via PPI co‐expression analysis and MCODE plug‐in in Cytoscape software. And we finally present a prognostic model via univariate and multivariate Cox regression analyses. 7 genes were included in the immune‐related genes signature, 2 of which were related to survival independently, namely CXCR3 and CXCL11. CXCR3 is known to have a key role in T‐cell trafficking to inflammatory sites and to tumours.^46^, ^47^ In our study, we found CXCR3 acts like protective factor in osteosarcoma. Lower expression of CXCR3 was associated with poor prognosis. We also found that CXCR3 was positively correlated with the infiltration of CD8 + T cell, follicular helper T cell and macrophage M1. Tang Yin et al reported similar results.^48^ Another study by Klatte Tobias et al reported that clear cell renal cell carcinoma patients with low CXCR3 expression (<30%) had a significantly worse prognosis.^49^ But some previous studies had reached different conclusions. Pradelli Emmanuelle et al demonstrated that to disrupt the CXCR3/CXCR3 ligand complexes with AMG487 could lead to a decrease in osteosarcoma lung metastasis.^50^ Reynders Nathan et al proposed the main reason for these controversial results was the crosstalk of the CXCR3 variants and their chemokine ligands within the tumour microenvironment.^51^ CXCR3‐A on malignant cells contributes to tumour growth and dissemination while CXCR3‐B has an anti‐proliferative effect. Chow et al revealed that successful PD‐1 immunotherapy requires expression of the CXCR3 chemokine receptor on CD8 + T cells.^52^ On the other hand, CXCR3‐endogenous ligands have been described to activate distinct signalling pathways, leading to specific cellular responses, such as CXCL9, CXCL11/CXCR3 axis. Interestingly, CXCL9 and CXCL11 were both included in our prognostic gene signature. This axis works primarily for immune cell migration, differentiation and activation. Immune cells show anti‐tumour effect against cancer cells through paracrine CXCL9, CXCL11/CXCR3 axis in tumour models. However, the autocrine signalling in cancer cells increases proliferation, angiogenesis and metastasis.^53^ Thus, it is promising to develop therapeutic targets by activating the paracrine axis, and inhibiting the autocrine axis. Unfortunately, there has been little research in osteosarcoma. In our validation, the relationships between CXCL9, CXCL11, CXCR3 and infiltration of immune cells were much similar. SAA1 coding for the major SAA isoform is expressed in trabecular and cortical bone fractions and bone marrow. Recently, SAA1 has been deemed to contribute to bone damage. Kovacevic Alenka et al reported high SAA1 levels in plasma of osteosarcoma patients.^54^ Beyond that, we failed to find more studies on osteosarcoma to investigate the other 3 genes in the signature. Even so, they still performed moderately in the validations of correlation with immune cell infiltration in both osteosarcoma and sarcoma data sets. In summary, this prognostic signature, based on differentially expressed genes in osteosarcoma, clearly reflected the infiltration of survival‐related immune cells and properly predicted clinical outcome.

## 5 CONCLUSION

In conclusion, we combined some algorithms to assess immune infiltration of osteosarcoma. In our analysis, macrophage (M0, M1 and M2) is the majority infiltration in osteosarcoma while CD8 + T cell, CD4 + naïve T cell and follicular helper T cell are related to survival outcome. We then explored a seven immune‐related gene prognostic signature, which was relatively credible to predict clinical outcome according to assessment of immune infiltration. Of importance, the potential relationship between CXCL9, CXCL11/CXCR3 and efficiency of immunotherapy should be further investigated. Our findings have provided novel insights that could demonstrate new targets of immunotherapy in osteosarcoma.

## CONFLICT OF INTEREST

The authors declare that they have no conflict of interest.

## AUTHOR CONTRIBUTION

**Yiyang Yu:** Data curation (lead); Formal analysis (equal); Investigation (lead); Methodology (lead); Resources (lead); Software (lead); Validation (lead); Visualization (lead); Writing‐original draft (lead); Writing‐review & editing (lead). **Hongliang Zhang:** Formal analysis (equal); Investigation (supporting); Methodology (supporting); Supervision (equal); Writing‐original draft (supporting); Writing‐review & editing (supporting). **Tingting Ren:** Project administration (supporting); Supervision (supporting); Validation (supporting); Writing‐original draft (supporting); Writing‐review & editing (supporting). **Yi Huang:** Supervision (supporting); Writing‐original draft (supporting); Writing‐review & editing (supporting). **Xin Liang:** Writing‐original draft (supporting); Writing‐review & editing (supporting). **Wei Wang:** Validation (supporting); Writing‐original draft (supporting); Writing‐review & editing (supporting). **Jianfang Niu:** Writing‐original draft (supporting); Writing‐review & editing (supporting). **Yu Han:** Conceptualization (supporting); Software (supporting); Validation (supporting); Visualization (supporting); Writing‐original draft (supporting); Writing‐review & editing (supporting). **Wei Guo:** Conceptualization (lead); Funding acquisition (lead); Project administration (lead); Supervision (lead).

## Supporting information

Fig S1

Fig S2

Fig S3

Fig S4

Fig S5

Fig S6

Table S1‐S4

## Data Availability

All data generated during this study are included in this published article. Publicly available data sets were analysed in this study, and these can be found in the Therapeutically Applicable Research To Generate Effective Treatments (https://ocg.cancer.gov/programs/target).
