# Supplementary figures and images for: Development of a prognostic gene signature based on an immunogenomic infiltration analysis of osteosarcoma
Source: J Cell Mol Med. 2020 Aug 21;24(19):11230–42. doi: 10.1111/jcmm.15687 (PMC7576232; doi:10.1111/jcmm.15687)

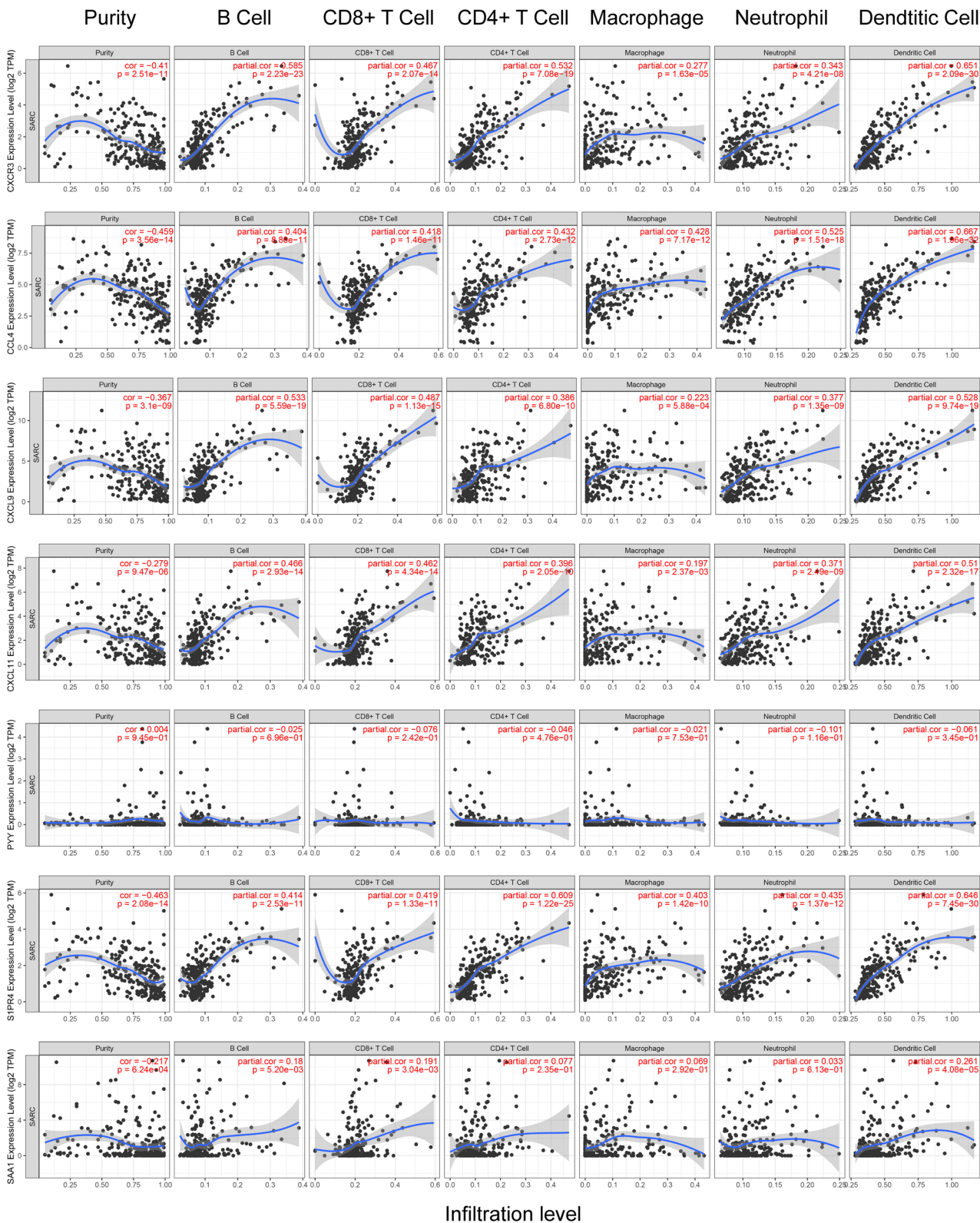

Infiltration level

Supplement: Supplementary file 1 — Fig S1 [file JCMM-24-11230-s001.pdf]

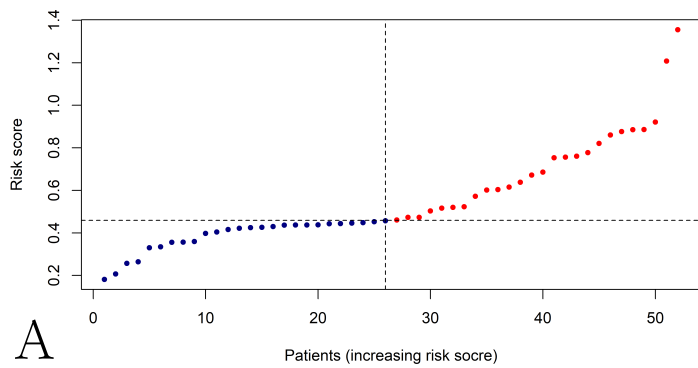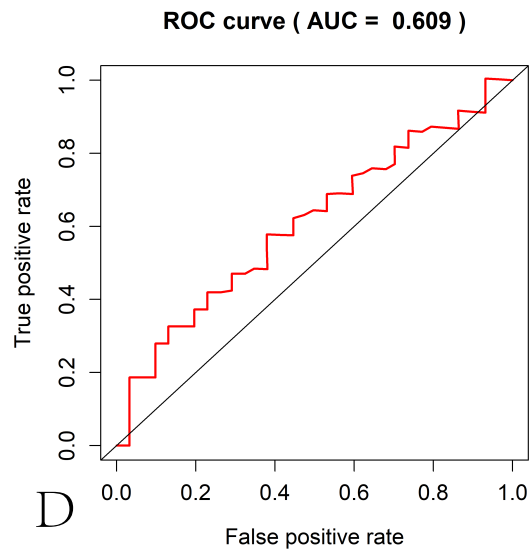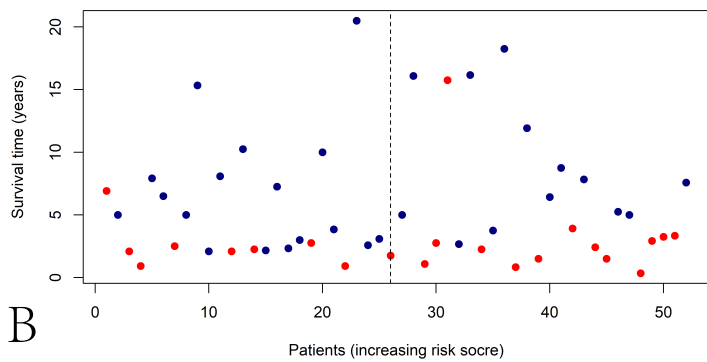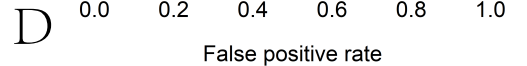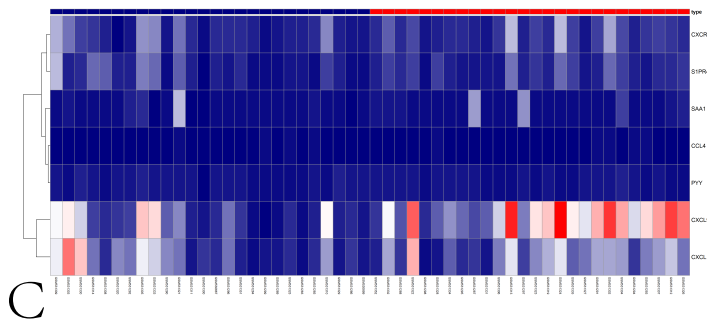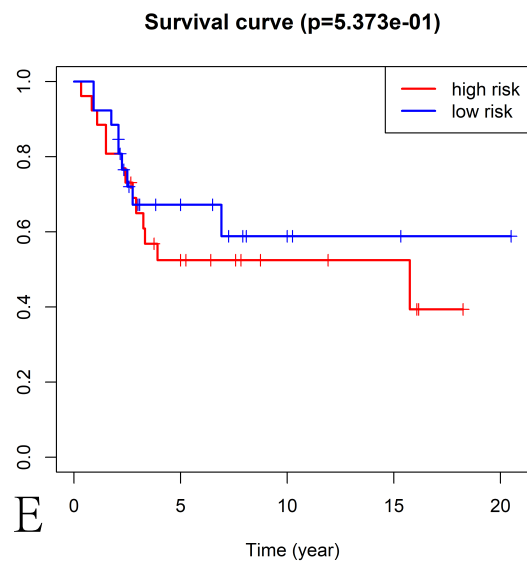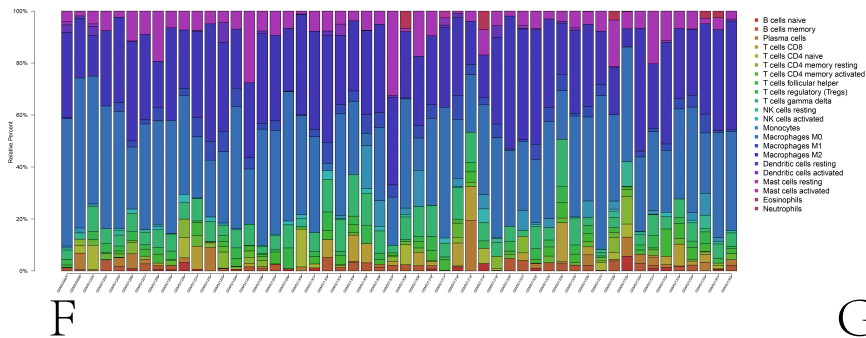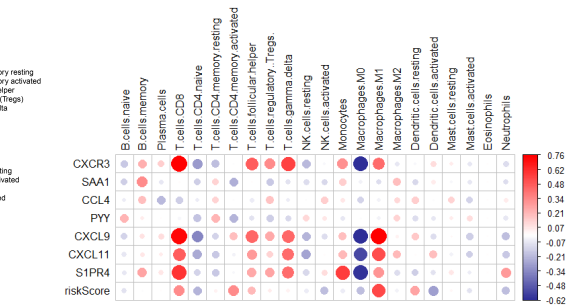

Supplement: Supplementary file 2 — Fig S2 [file JCMM-24-11230-s002.pdf]

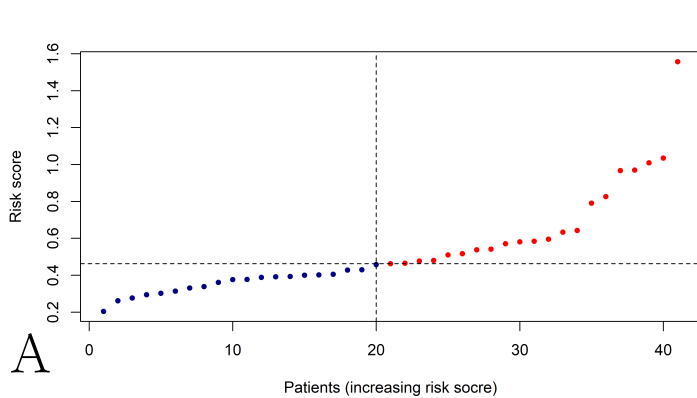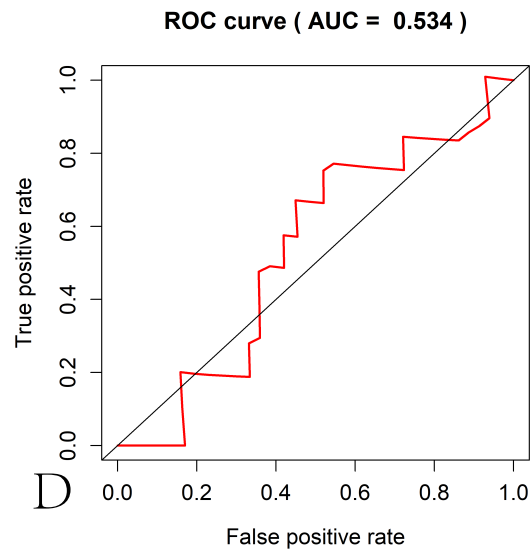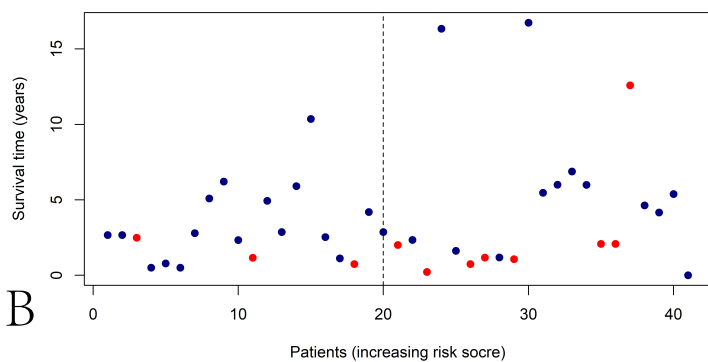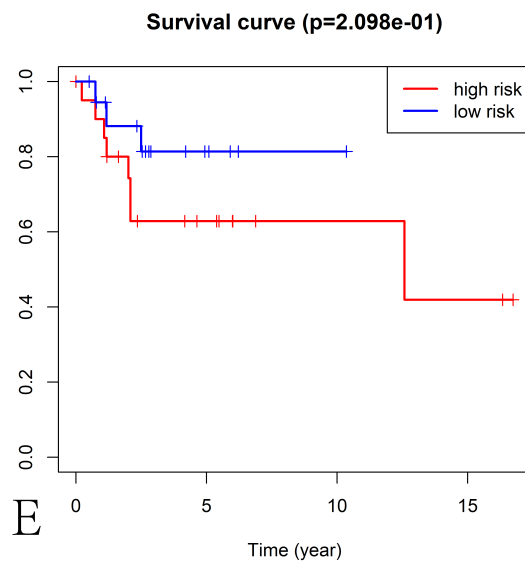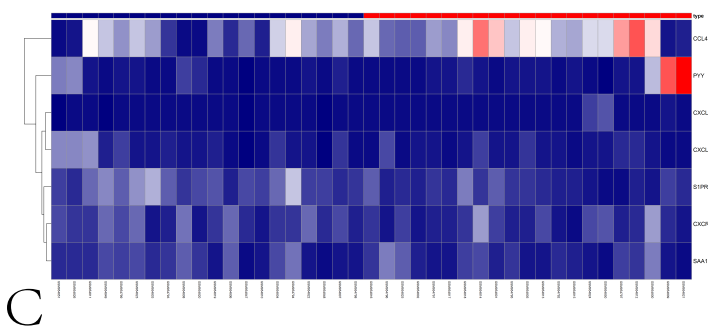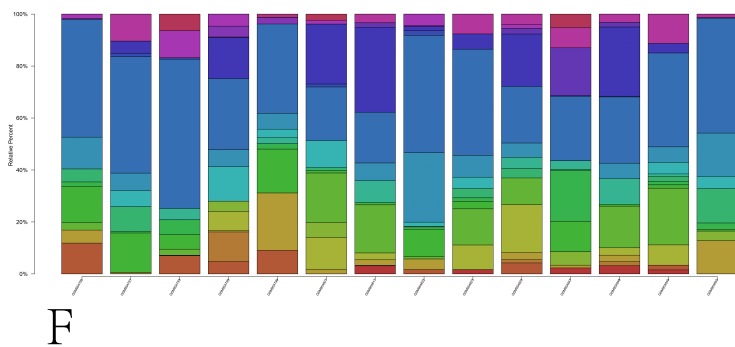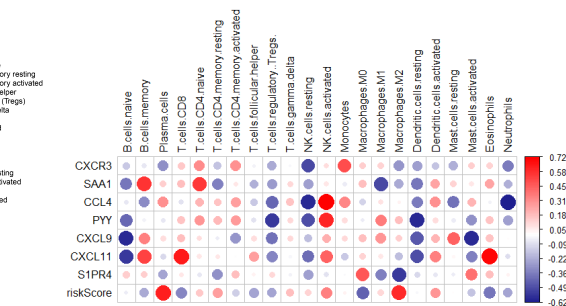

Supplement: Supplementary file 3 — Fig S3 [file JCMM-24-11230-s003.pdf]

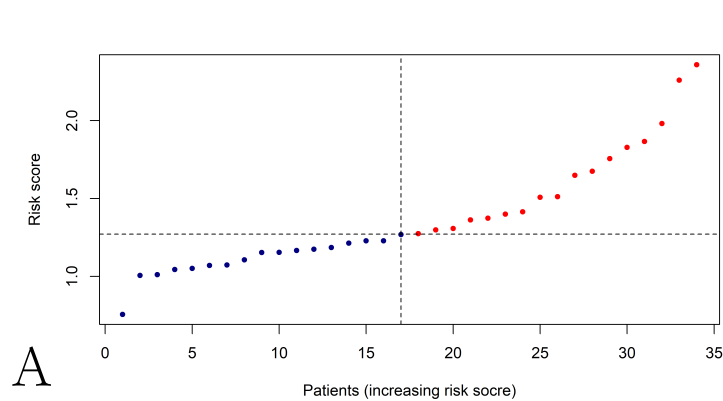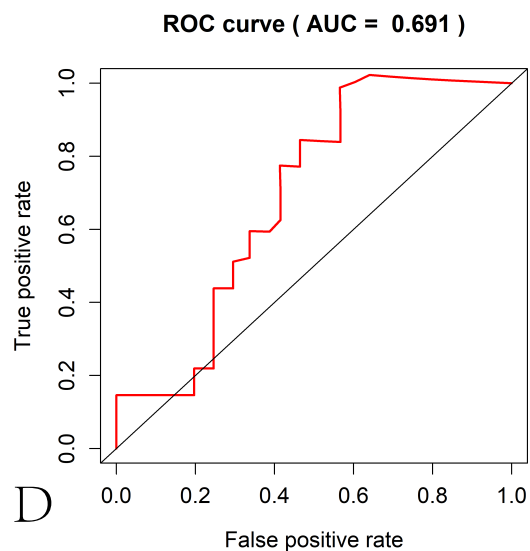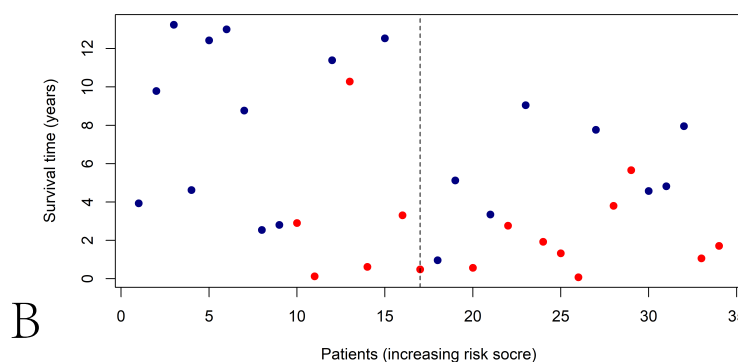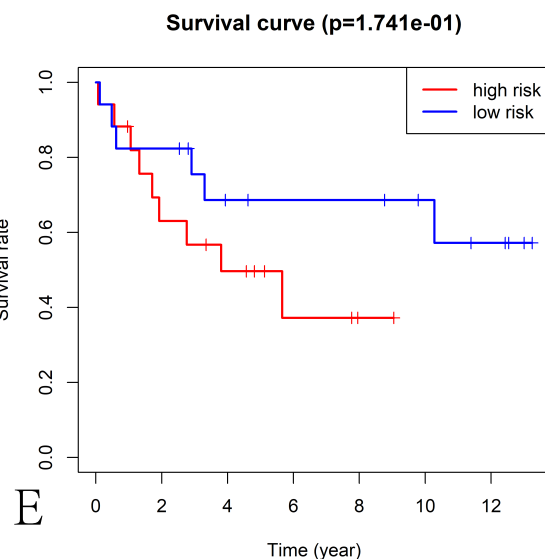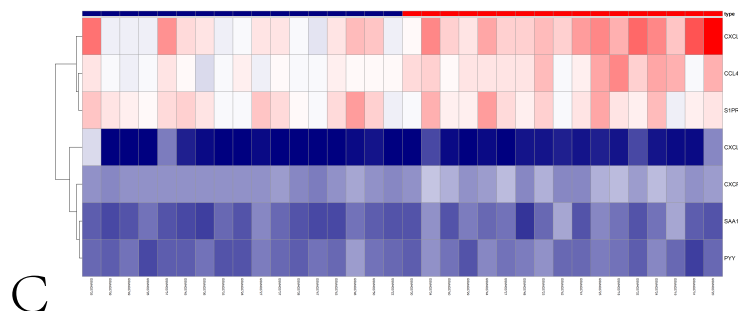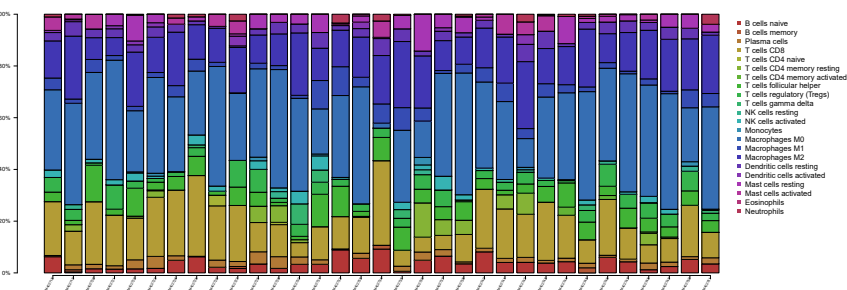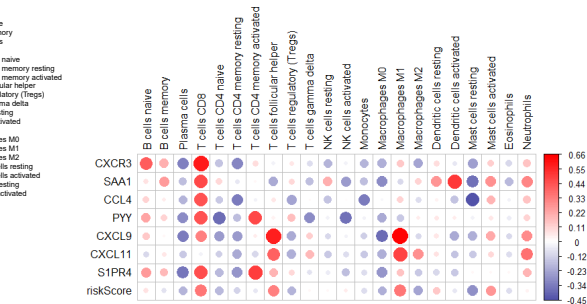

Supplement: Supplementary file 4 — Fig S4 [file JCMM-24-11230-s004.pdf]

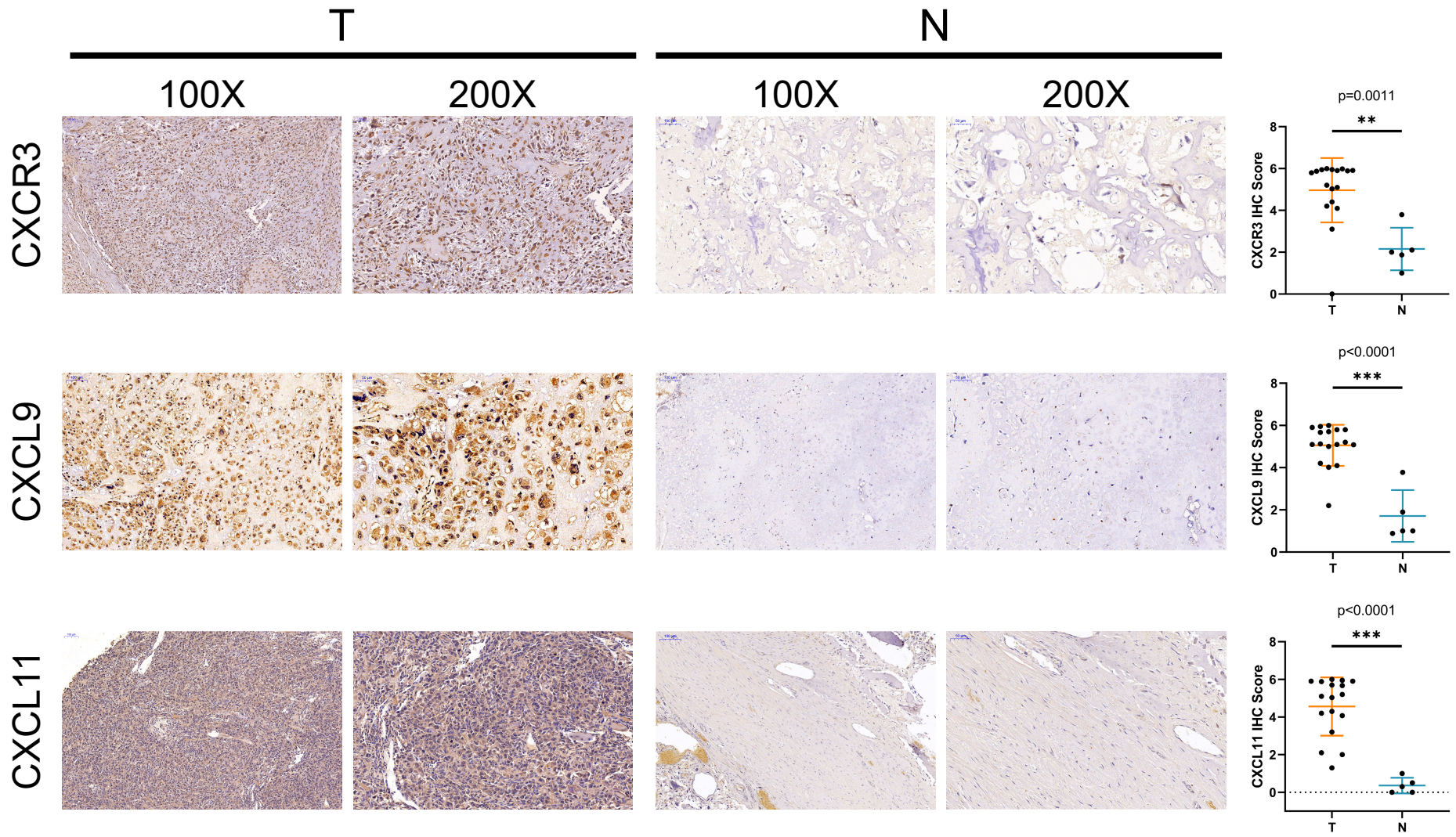

Supplement: Supplementary file 5 — Fig S5 [file JCMM-24-11230-s005.pdf]

M

100X

200X

CXCR3

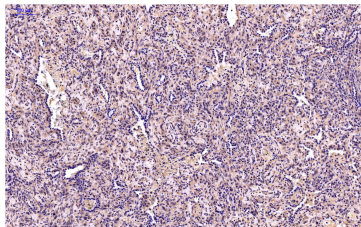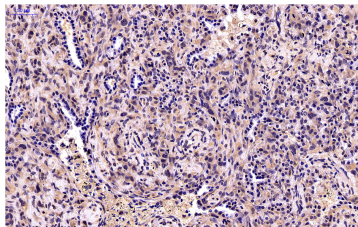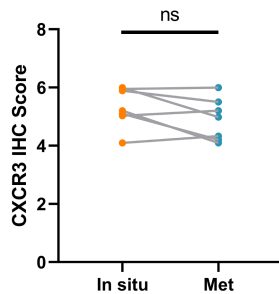

CXCL9

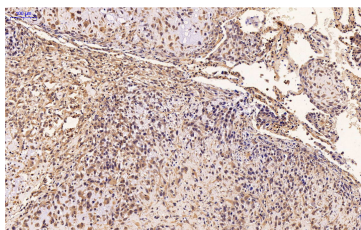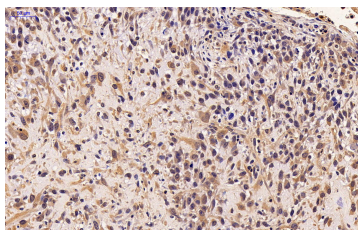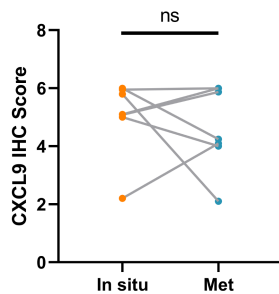

CXCL11

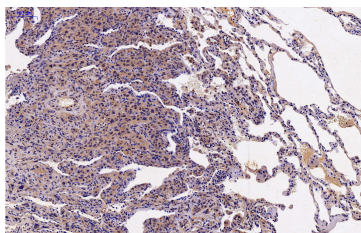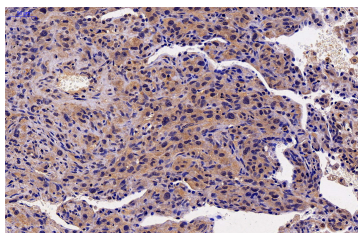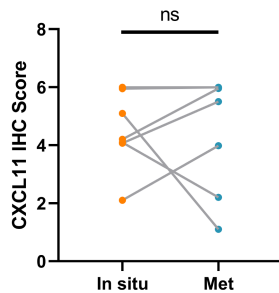

Supplement: Supplementary file 6 — Fig S6 [file JCMM-24-11230-s006.pdf]
