# Supplementary material for: Development of a prognostic gene signature based on an immunogenomic infiltration analysis of osteosarcoma
Source: J Cell Mol Med. 2020 Aug 21;24(19):11230–42. doi: 10.1111/jcmm.15687 (PMC7576232; doi:10.1111/jcmm.15687)
Supplement: Supplementary file 7 — Table S1‐S4 [file JCMM-24-11230-s007.docx]

**Tables**

**Table S1. Detailed clinical information of TARGET-OS dataset**

| **Characteristics** | | **TARGET dataset** |
| --- | --- | --- |
| Gender | Male | 58 |
|  | Female | 40 |
| Survival Status | Live | 45 |
|  | Death | 50 |
| Metastasis | Metastasis | 24 |
|  | None-Metastasis | 74 |
| First Event | Censored | 13 |
|  | Death | 4 |
|  | None | 33 |
|  | Progression/Relapse | 46 |
| Necrosis stage | Stage 1/2 | 33 |
|  | Stage 3/4 | 19 |
| 5-year survival | >5 years | 32 |
|  | <5 years | 45 |

**Table S2. Function roles of 13 hub genes**

| **No.** | **Gene** | **Full name** | **Function** |
| --- | --- | --- | --- |
| 1 | CXCL11 | C-X-C motif chemokine ligand 11 | Induces calcium release in activated T-cells. |
| 2 | CXCR3 | C-X-C motif chemokine receptor 3 | Receptor for the C-X-C chemokine CXCL9, CXCL10 and CXCL11 |
| 3 | CXCR6 | C-X-C motif chemokine receptor 6 | Receptor for the C-X-C chemokine CXCL16. |
| 4 | S1PR4 | sphingosine-1-phosphate receptor 4 | Receptor for the lysosphingolipid sphingosine 1-phosphate (S1P) |
| 5 | CXCL2 | C-X-C motif chemokine ligand 2 | Produced by activated monocytes and neutrophils |
| 6 | SAA1 | serum amyloid A1 | Major acute phase protein. |
| 7 | CXCL10 | C-X-C motif chemokine ligand 10 | Pro-inflammatory cytokine involved in a wide variety of processes |
| 8 | PYY | peptide YY | Inhibits exocrine pancreatic secretion, and inhibitis jejunal and colonic mobility |
| 9 | CCL4 | C-C motif chemokine ligand 4 | Monokine with inflammatory and chemokinetic properties |
| 10 | CCR9 | C-C motif chemokine receptor 9 | Receptor for chemokine SCYA25/TECK |
| 11 | CXCL9 | C-X-C motif chemokine ligand 9 | Cytokine that affects the growth, movement, or activation state of cells |
| 12 | SSTR3 | somatostatin receptor 3 | Receptor for somatostatin-14 and -28 |
| 13 | C3 | complement C3 | Plays a central role in the activation of complement system. |

**Table S3. univariate Cox analysis result**

| gene | HR | z | pvalue |
| --- | --- | --- | --- |
| CXCR3 | 0.825232 | -2.21876 | 0.026503 |
| SSTR3 | 0.96582 | -0.25129 | 0.801587 |
| SAA1 | 1.070094 | 1.17742 | 0.239028 |
| CCL4 | 0.941591 | -0.63928 | 0.52264 |
| PYY | 1.106809 | 0.886724 | 0.375228 |
| CCR9 | 1.038687 | 0.414461 | 0.678536 |
| CXCL9 | 0.974373 | -0.35501 | 0.72258 |
| CXCL11 | 0.802579 | -2.66447 | 0.007711 |
| C3 | 0.987386 | -0.14529 | 0.884482 |
| CXCL2 | 1.093839 | 1.207413 | 0.227273 |
| S1PR4 | 0.839257 | -1.72236 | 0.085004 |
| CXCL10 | 0.925169 | -1.06611 | 0.286376 |
| CXCR6 | 0.828432 | -1.93823 | 0.052595 |

**Table S4** **Antibodies used in this study**

| **Antibody name** | **Corporation name** | **Catalog No.** | **Dilution ratio** |
| --- | --- | --- | --- |
| CXCR3 | proteintech | 26756-1-AP | 1:200 |
| CXCL9 | proteintech | 22355-1-AP | 1:200 |
| CXCL11 | proteintech | 10707-1-AP | 1:200 |
| Goat anti-rabbit IgG | Thermo fisher | 31460 | 1:2000 |
